# mRNALocator-imb: an imbalance-tolerant ensemble framework integrating random forest and transformer for mRNA subcellular localization prediction

**DOI:** 10.3389/fgene.2026.1861282

**Published:** 2026-05-20

**Authors:** Jinbo Hu, Haibin Liu, Lanzhong Wang, Hao Wu

**Affiliations:** 1 School of Airspace Science and Engineering, Shandong University, Weihai, Shandong, China; 2 School of Software, Shandong University, Jinan, Shandong, China; 3 Shenzhen Research Institute, Shandong University, Shenzhen, Guangdong, China; 4 School of Foreign Languages and Literature, Shandong University, Jinan, Shandong, China

**Keywords:** adaptive synthetic sampling, deep learning frameworks, imbalanced dataset, label-distribution-aware margin, mRNA subcellular localization

## Abstract

The subcellular localization of mRNAs determines the spatial context of protein translation and plays a critical role in the precise regulation of protein function. However, existing computational approaches for predicting eukaryotic mRNA localization are often limited by inadequate exploitation of sequence-derived information and suboptimal performance under highly imbalanced data distributions. To address these challenges, we propose mRNALocator-imb, an imbalance-aware ensemble framework for mRNA subcellular localization prediction. The proposed model integrates physicochemical property–based features with k-mer frequency vectors, and adopts a hybrid architecture that combines Random Forest and Transformer networks to capture both global sequence patterns and contextual dependencies. To explicitly mitigate class imbalance, Random Oversampling and Label-Distribution-Aware Margin (LDAM) loss are incorporated during Transformer training, while the Synthetic Minority Over-sampling Technique (SMOTE) is employed to rebalance the data for Random Forest learning. Extensive experiments demonstrate that mRNALocator-imb consistently outperforms conventional machine learning methods and state-of-the-art predictors, particularly in scenarios characterized by severe class imbalance. Overall, this study presents a robust and generalizable framework for sequence-based subcellular localization prediction, with broad applicability to other imbalanced learning problems in bioinformatics.

## Introduction

Messenger RNA (mRNA) is a single-stranded RNA molecule that transmits genetic information from DNA to ribosomes, where it serves as the template for protein synthesis. In eukaryotic cells, mRNAs are not randomly distributed; rather, their intracellular localization is governed by highly regulated and selective processes. This spatial organization enables localized translation at specific subcellular sites, thereby ensuring precise spatiotemporal control of protein production and facilitating appropriate cellular responses to both intrinsic and extrinsic stimuli (Mili and Macara, 2009; Holt and Bullock, 2009; Benoit Bouvrette et al., 2018).

Historically, mRNA localization and translational regulation were considered specialized mechanisms affecting only a limited subset of transcripts, primarily to confine gene expression to polarized or asymmetric cellular regions (Jansen, 2001). However, accumulating evidence has established that mRNA subcellular localization is a pervasive regulatory phenomenon. It is implicated in a wide range of biological processes, including cell polarity, cell migration, embryonic development, and asymmetric cell division, as well as in the pathogenesis of complex diseases such as Alzheimer’s disease and cancer (Engel et al., 2020; Katz et al., 2012; Kejiou and Palazzo, 2017; Liu et al., 2019). A comprehensive understanding of mRNA localization is therefore essential for elucidating intracellular information flow, uncovering disease-associated molecular mechanisms, and advancing therapeutic development.

Early investigations of mRNA localization relied predominantly on experimental techniques. Methods such as RNA fluorescence *in situ* hybridization (RNA-FISH) (Lawrence and Singer, 1986), CRISPR LiveFISH (Wang et al., 2019), and INSIGHT (Ren et al., 2020) enabled direct visualization of RNA molecules within cells. With the rapid development of high-throughput sequencing technologies, RNA sequencing (RNA-seq) has provided transcriptome-wide insights into RNA abundance and function. More recently, sequencing-based approaches, including subRNA-seq (Mayer and Churchman, 2017), FISSEQ (Lee et al., 2014), and APEX-seq (Fazal et al., 2019), have enabled large-scale characterization of RNA spatial distribution across subcellular compartments.

Despite their utility, these experimental approaches present several inherent limitations. They typically require specialized instrumentation and technical expertise, involve labor-intensive procedures, and incur substantial financial and time costs. Consequently, their scalability is restricted, limiting their applicability in large-scale or high-throughput studies.

These limitations have motivated the development of computational approaches for predicting RNA subcellular localization. By leveraging bioinformatics and machine learning techniques, such methods offer efficient, cost-effective alternatives that can extract biologically meaningful patterns from large-scale datasets and enable systematic prediction of RNA localization and function.

Recent years have witnessed notable progress in this field. RNATracker (Yan et al., 2019), the first deep learning–based predictor for mRNA subcellular localization, integrates convolutional neural networks, bidirectional long short-term memory (BiLSTM), and self-attention mechanisms to enhance predictive performance. However, it relies exclusively on primary-sequence one-hot encoding without incorporating physicochemical properties, multi-scale k-mer representations, or handcrafted biologically informative descriptors, thereby limiting exploitation of intrinsic sequence characteristics. Subsequently, Zhang et al. proposed iLoc-mRNA (Zhang et al., 2021), an SVM-based predictor that utilizes only 9-mer composition features combined with a two-step feature selection strategy. Although it achieved high accuracy on human datasets, the model depends on a single fixed k-mer length and neglects short-range motifs, sequence-order effects, and global contextual dependencies, resulting in incomplete utilization of sequence information. To extend applicability across species, Garg et al. developed mRNALoc (Garg et al., 2020), which employs only PseKNC features as input to an SVM classifier. Despite its broader species coverage, mRNALoc relies on a single pseudo-composition feature type and lacks complementary encodings, such as physicochemical properties, mismatch-tolerant representations, and reverse-complement k-mers, thereby failing to comprehensively capture multifaceted sequence patterns.

Although these methods have achieved encouraging performance, each underutilizes sequence information in distinct ways: RNATracker lacks structured feature engineering, iLoc-mRNA relies on an overly constrained fixed-length k-mer representation, and mRNALoc depends on a single feature modality. In addition, most existing approaches show limited robustness to class imbalance and provide insufficient biological interpretability. These limitations motivated the development of a more comprehensive predictive framework.

To address these challenges, this study proposes a comprehensive framework that simultaneously enhances prediction accuracy, robustness to class imbalance, and biological interpretability. Specifically, we employ a rigorous feature engineering strategy that integrates multiple complementary sequence-derived representations to capture diverse characteristics of mRNA sequences. We further combine advanced machine learning and deep learning architectures with imbalance-aware optimization strategies to construct a robust predictive model. In addition, we incorporate feature importance and interpretability analyses to identify key sequence-derived biomarkers and to elucidate their biological relevance from both model-driven and sequence-level perspectives.

By jointly improving predictive performance, imbalance tolerance, and interpretability, this work provides deeper insights into the mechanisms underlying mRNA subcellular localization. The proposed framework not only advances the computational prediction of RNA localization but also offers a generalizable strategy for tackling imbalanced learning problems in bioinformatics, thereby contributing valuable tools and biological insights for future research.

## Materials and methods

### Datasets

The dataset used in this study was obtained from mRNALoc, a curated repository of mRNA subcellular localization annotations (Garg et al., 2020). In total, 14,909 mRNA sequences were collected and categorized into five subcellular compartments: cytoplasm (6,376), endoplasmic reticulum (1,426), extracellular region (855), mitochondria (421), and nucleus (5,831).

For model development and evaluation, the dataset was partitioned into a training set, a weighted set, and an independent test set using a stratified sampling strategy with an approximate ratio of 5:1:1. This partitioning scheme was designed to provide sufficient training samples for robust model fitting, a dedicated weighted set for optimizing the ensemble strategy without information leakage, and an adequately sized independent test set for unbiased and reliable performance assessment. Stratified sampling was applied to preserve class distributions across all subsets, thereby reducing sampling bias and ensuring stable evaluation under class-imbalanced conditions. Detailed data partitioning and class distributions are presented in Table 1.

**TABLE 1:** Subcellular location distribution of mRNA sequences in the datasets.

Location	Training	Weighted	Test	Total
Cytoplasm	4,554	910	912	6,376
Endoplasmic_reticulum	1,018	203	205	1,426
Extracellular_region	610	122	123	855
Mitochondria	300	60	61	421
Nucleus	4,165	833	833	5,831

### Feature encoding schemes

Feature fusion has been shown to substantially enhance predictive performance in sequence-based learning tasks (Wu et al., 2022; Zhang et al., 2022a; Zhang et al., 2022b). In this study, five complementary feature encoding schemes, including TPCP, Mismatch, RCKmer, PseKNC, and k-mer, were employed to capture diverse characteristics of mRNA sequences.

Specifically, TPCP, Mismatch, RCKmer, and PseKNC features were used as inputs to the Random Forest classifier, whereas k-mer representations were utilized for the Transformer-based model. These encoding schemes provide complementary perspectives, integrating physicochemical properties, sequence composition, and motif-level information. Detailed formulations and biological interpretations are provided in Supplementary Material S1.

### Overview of the mRNALocator-imb framework

**FIGURE 1:**
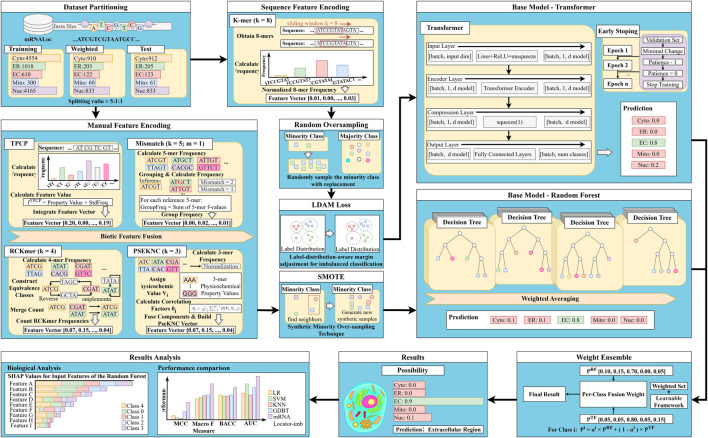
Overview of the mRNALocator-imb framework.

As illustrated in Figure 1, mRNALocator-imb is a heterogeneous deep ensemble framework designed to predict mRNA subcellular localization directly from nucleotide sequences through multi-scale feature integration. The framework establishes an end-to-end mapping from raw sequences to localization labels, eliminating the need for manual feature post-processing.

The pipeline begins with raw mRNA sequences in FASTA format, which are processed through two parallel encoding branches. The first branch generates handcrafted feature representations (TPCP, Mismatch, RCKmer, and PseKNC), capturing biologically interpretable properties such as nucleotide composition, physicochemical characteristics, and motif patterns (e.g., G-rich or C-rich elements and specific trinucleotide motifs). These features enable transparent, interpretable modeling of sequence–localization relationships.

In parallel, the second branch employs k-mer encoding to construct high-dimensional frequency vectors that capture intrinsic compositional patterns and local sequence dependencies. This data-driven representation complements handcrafted descriptors by preserving fine-grained sequence information.

To mitigate the limitations of single-modality representations, the dual-branch architecture integrates both biologically interpretable and data-driven features, providing a comprehensive characterization of mRNA sequences.

To address class imbalance, dedicated strategies were introduced prior to model training. Specifically, Random Oversampling and the LDAM loss were employed during Transformer training, whereas SMOTE was applied to rebalance the handcrafted feature space used for Random Forest classification.

At the decision level, a heterogeneous ensemble strategy is employed. A Random Forest classifier generates class probability estimates $P^{RF}$ from handcrafted features, while a Transformer network produces complementary predictions $P^{TF}$ from k-mer representations. These outputs are integrated *via* a weighted ensemble module with learnable parameters, enabling adaptive fusion of complementary predictive signals. This design enhances both accuracy and generalization across diverse localization categories.

### Base classifier construction

Random Forest (RF) models have been widely applied in bioinformatics due to their robustness and effectiveness in handling structured biological features (Yang et al., 2024; Yang et al., 2023; Ye et al., 2023). In this study, an RF classifier was constructed using fused handcrafted features to capture interpretable sequence characteristics.

However, handcrafted features alone may fail to fully preserve intrinsic nucleotide composition and local motif dependencies. To address this limitation, k-mer–based sequence representations were incorporated. k-mer analysis segments sequences into overlapping substrings of length k using a sliding window (step size = 1), and constructs normalized frequency vectors representing the occurrence of each k-mer type. For the Transformer branch, only k-mers with k = 8 were utilized as input features. In contrast, k values of 3, 4, and 5 were not used in the Transformer branch, but were specifically adopted for handcrafted feature extraction in the Random Forest branch: k = 5 for Mismatch, k = 4 for RCKmer, and k = 3 for PseKNC (Vinga et al., 2003; Li et al., 2014).

To further model long-range dependencies among sequence elements, the k-mer feature vectors were processed using a Transformer network. This architecture enables effective learning of contextual relationships and higher-order compositional patterns within mRNA sequences, thereby complementing the RF-based feature learning process (Xiang et al., 2023).

### Weighted ensemble integration

The final prediction of mRNALocator-imb is obtained through a weighted averaging ensemble of the RF and Transformer models. To prevent information leakage, an independent weighted set was reserved exclusively for learning ensemble parameters.

Both base models were first trained on the training set. For the Transformer, the training data were further split into training and validation subsets (9:1 ratio), and model optimization was performed using ten-fold cross-validation combined with early stopping.

Subsequently, the weighted set was used to optimize the ensemble weights, enabling adaptive calibration of the contributions from each model. This strategy improves predictive robustness and ensures unbiased integration of heterogeneous learners.

### Data imbalance handling strategy

Class imbalance is a pervasive challenge in bioinformatics and can significantly degrade model performance, particularly for minority classes. In mRNA localization datasets, substantial disparities in class sizes can lead to biased decision boundaries and reduced sensitivity for underrepresented categories.

To address this issue, we adopted a hybrid imbalance-handling strategy combining data-level and algorithm-level approaches. For the RF model, the Synthetic Minority Over-sampling Technique (SMOTE) (Tao et al., 2024) was employed. SMOTE generates synthetic minority samples by interpolating between neighboring instances, thereby expanding the minority feature space and improving decision boundary representation.

For the Transformer model, a dual strategy was implemented. Random Oversampling was applied at the data level to balance class distributions, while the Label-Distribution-Aware Margin (LDAM) loss was introduced at the optimization level to enhance minority-class learning. LDAM assigns larger decision margins to minority classes, encouraging improved discrimination of underrepresented categories.

The LDAM loss (Equation 1) is defined as:

$$L_{\text{LDAM}}=-\log\frac{\exp\left(z_{y}-\Delta_{y}\right)}{\exp\left(z_{y}-\Delta_{y}\right)+\sum_{j\neq y}\exp\left(z_{j}\right)}$$ (1)

where $z_{y}$ denotes the logit of the true class and $z_{j}$ represents the logits of other classes. The class-dependent margin (Equation 2) is defined as:

$$\Delta_{y}=\frac{C}{n_{y}^{1/4}},y\in \left\{1,\cdots ,K\right\}$$ (2)

where $n_{y}$ is the number of training samples in class y , K is the total number of classes, and C is a scaling hyperparameter.

### Hyperparameter optimization

Hyperparameter tuning is critical for achieving optimal model performance and preventing overfitting. In this study, Bayesian optimization combined with three-fold stratified cross-validation was employed to jointly optimize the RF and Transformer models.

The Matthews Correlation Coefficient (MCC) was used as the optimization objective due to its robustness in evaluating imbalanced classification tasks. This approach enables systematic exploration of the hyperparameter space while ensuring balanced performance across classes.

Key hyperparameters, including RF structural parameters and Transformer architecture configurations, were optimized with respect to ensemble performance. The final selected hyperparameters are summarized in Table 2.

**TABLE 2:** Hyperparameter optimization results.

Hyperparameters	Search range	Optimal value
Number of trees in a random forest	[100, 150, 200]	200
Maximum depth of random forest	[Unlimited, 10, 20, 30]	30
Minimum sample split for random forest	[2, 5, 8, 10]	2
Minimum sample leaf for random forest	[1, 2, 5, 8]	1
Max features for random forest	['auto'^1^, 'sqrt'^2^, 'log2'^3^]	'sqrt'
Bootstrap sampling for random forest	[No, yes]	No
Model dimension of transformer	[16, 32, 64]	16
Number of heads in transformer	[2, 4]	2
Number of encoder layers in transformer	[1, 2, 3]	2
Feedforward dimension of transformer	[32, 64, 128]	32
Dropout rate of transformer	[0.0, 0.1, 0.2]	0.1

## Results

### Ten-fold cross-validation on the training set

Ten-fold cross-validation is a standard strategy for evaluating model generalization, stability, and robustness. In this procedure, the training dataset is partitioned into ten equal subsets; in each iteration, nine subsets are used for training and the remaining subset for validation. This approach maximizes data utilization and reduces variance associated with a single data split, thereby providing a reliable estimate of model performance.

To assess the effectiveness of mRNALocator-imb, we performed ten-fold cross-validation on the training set using Macro F-measure, balanced accuracy (BACC), and area under the ROC curve (AUC) as evaluation metrics (definitions provided in Supplementary Material S2).

As summarized in Table 3, mRNALocator-imb demonstrates stable and competitive performance across all folds, achieving mean values of 0.7175 (Macro F-measure), 0.6902 (BACC), and 0.9040 (AUC). The relatively low variance across folds indicates strong robustness and generalization capability in mRNA subcellular localization prediction.

**TABLE 3:** Ten-fold cross-validation results on the training set.

Ten-fold cross validation	Macro F measure	BACC	AUC
Fold-1	0.7040	0.6863	0.8760
Fold-2	0.7196	0.6913	0.9286
Fold-3	0.7261	0.6913	0.8847
Fold-4	0.6961	0.6684	0.8735
Fold-5	0.7161	0.6889	0.8924
Fold-6	0.7466	0.7127	0.9279
Fold-7	0.7300	0.6983	0.9319
Fold-8	0.7264	0.6970	0.9294
Fold-9	0.6799	0.6607	0.8737
Fold-10	0.7303	0.7067	0.9223
Mean	0.7175	0.6902	0.9040

### Validation of data imbalance handling strategies

To systematically evaluate the effectiveness of the proposed imbalance-handling strategies, we conducted comparative analyses from two perspectives: (i) data-level resampling methods for traditional machine learning models and (ii) loss function design for deep learning models.

### Evaluation of resampling strategies for random forest

We compared five representative resampling techniques, including the Synthetic Minority Over-sampling Technique (SMOTE) (Tao et al., 2024), the Adaptive Synthetic Sampling (ADASYN) algorithm (Munshi, 2024), Random Oversampling, Random Undersampling, and the NearMiss algorithm (Wang et al., 2022), along with a no-resampling baseline. All Random Forest models were trained under identical conditions to ensure fair comparison.

**FIGURE 2:**
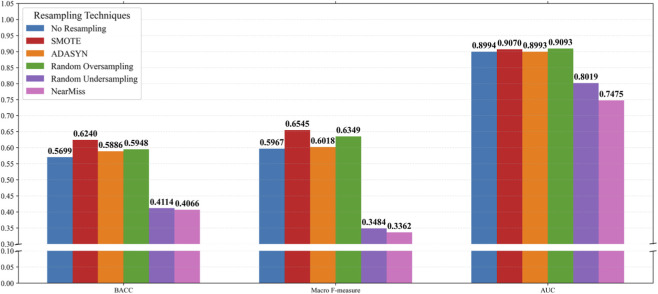
Performance comparison of different resampling techniques on Random Forest.

As shown in Figure 2, the resampling strategy significantly affects model performance. Among all methods, SMOTE achieved the best overall results, followed by Random Oversampling and the no-resampling baseline, while undersampling-based methods (NearMiss and Random Undersampling) exhibited substantially inferior performance. These findings indicate that SMOTE effectively enhances minority-class representation without losing majority-class information, and was therefore selected for the Random Forest sub-model.

### Evaluation of resampling strategies for transformer

We further conducted analogous experiments on the Transformer model. As summarized in Table 4, Random Oversampling achieved the best overall performance, yielding the highest ACC, MCC, Macro F-measure, and Precision. Although its AUC was slightly lower than that of certain alternatives, it remained within a high-performance range.

**TABLE 4:** Performance comparison of different resampling techniques on transformer.

Resampling method	ACC	MCC	Precision	Recall	Macro F measure	BACC	AUC
No resampling	0.7427	0.5978	0.6864	0.5942	0.6197	0.5942	0.8616
SMOTE	0.7558	0.6130	0.7293	0.593	0.6325	0.5930	0.854
ADASYN	0.7442	0.5994	0.7113	0.5972	0.6299	0.5972	0.8614
Random oversampling	0.7581	0.6150	0.7737	0.5948	0.6391	0.5948	0.8492
NearMiss	0.5166	0.2799	0.5082	0.4078	0.3917	0.4078	0.7407
Random undersampling	0.5548	0.3474	0.6096	0.4751	0.4571	0.4751	0.7649

In contrast, SMOTE ranked second, while ADASYN showed moderate performance without clear advantages. Undersampling methods again performed poorly, confirming that removing the majority-class samples leads to loss of critical information.

These results highlight that the optimal resampling strategy is model-dependent: SMOTE is more suitable for Random Forest, whereas Random Oversampling is better aligned with the Transformer architecture.

### Comparative analysis of resampling strategies

Across both model types, oversampling methods consistently outperformed undersampling approaches. This is primarily because oversampling enhances minority-class representation while preserving the full information content of the dataset, whereas undersampling discards potentially informative majority-class samples.

Notably, the performance differences between SMOTE and Random Oversampling can be attributed to their underlying mechanisms. SMOTE generates synthetic samples through interpolation, which improves decision boundary learning in feature-based models such as Random Forest. In contrast, Random Oversampling avoids introducing synthetic noise, making it more compatible with deep learning models that are capable of learning complex feature representations directly from replicated samples.

### Evaluation of loss functions for transformer

To further improve imbalance robustness in deep learning, we evaluated five loss functions: cross-entropy (CE) (Mao et al., 2023), focal loss (Lin et al., 2017), weighted cross-entropy (WCE) (Phan and Yamamoto, 2024), the Lovász-softmax (Berman et al., 2018), and the Label-Distribution-Aware Margin (LDAM) (Cao et al., 2019).

**FIGURE 3:**
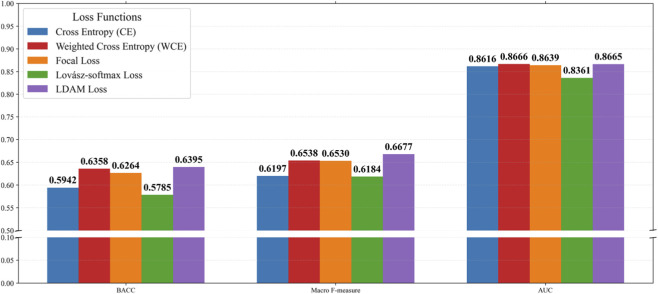
Performance comparison of different loss functions.

As shown in Figure 3, LDAM achieved the best overall performance, yielding the highest Macro F-measure and BACC while maintaining competitive AUC. In contrast, standard CE loss exhibited stable but inferior performance, and Lovász-softmax showed the weakest results.

These findings demonstrate that LDAM effectively enhances minority-class discrimination by incorporating class-dependent margins, making it particularly suitable for imbalanced mRNA localization tasks.

### Performance comparison on the independent test set

To further validate the proposed framework, we compared mRNALocator-imb with three representative methods, including RNATracker, mRNALoc, and iLocmRNA, on an independent test set.

**FIGURE 4:**
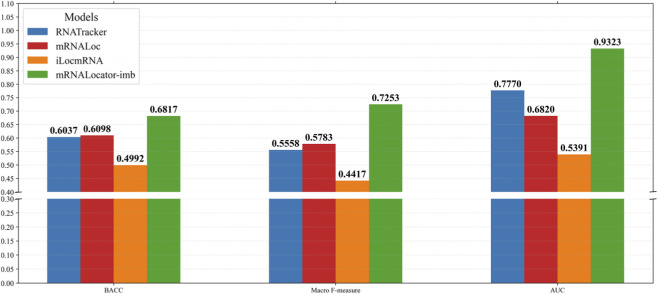
Performance comparison of mRNALocator-imb and existing methods.

As illustrated in Figure 4, mRNALocator-imb consistently outperforms all baseline methods across all evaluation metrics. Specifically, it achieves a Macro F-measure of 0.7253, substantially exceeding mRNALoc (0.5783), RNATracker (0.5558), and iLocmRNA (0.4417). Similarly, it attains superior BACC (0.6817) and AUC (0.9323), indicating strong discriminative ability and balanced performance across classes.

These results demonstrate that the proposed framework provides significant improvements over existing methods. The performance gains can be attributed to the integration of multi-scale feature representations, the heterogeneous ensemble architecture, and the effective imbalance-aware optimization strategies.

### Analysis of feature contribution and dependencies

To interpret model behavior and identify key determinants of mRNA localization, we applied SHapley Additive exPlanations (SHAP) to quantify feature contributions exclusively for the Random Forest branch. Interpretability analysis of the Transformer branch was not performed and is beyond the scope of this study.

Feature importance was computed as the mean absolute SHAP value (Equation 3) across all samples:

$$FI_{j}=\frac{1}{n}\sum_{i=1}^{n}\;\left|\varphi_{j}^{\left(i\right)}\right|$$ (3)

where $\varphi_{j}^{\left(i\right)}$ represents the SHAP value of feature j for sample i .

**FIGURE 5:**
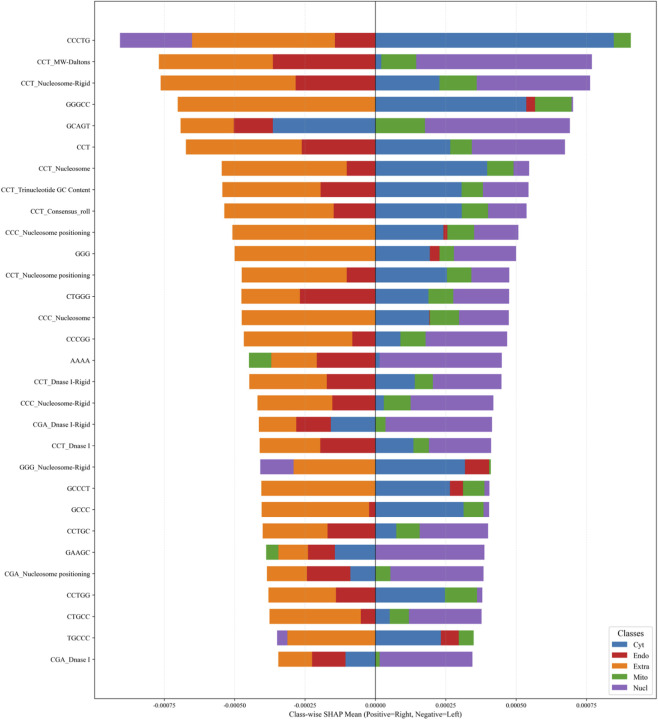
SHAP analysis of feature importance for the Random Forest branch.

As shown in Figure 5, the top-ranked features include CCCTG, CCT_MW-Daltons, and CCT_Nucleosome-Rigid, followed by GGGCC, GCAGT, and CCT. These features predominantly belong to two categories: (i) C/G-enriched k-mer sequence motifs and (ii) functional descriptors associated with the CCT trinucleotide motif.

Importantly, these features exhibit strong class-specific effects. For example, CCCTG shows a positive contribution to cytoplasmic localization but negative contributions to extracellular and nuclear compartments, whereas CCT_MW-Daltons strongly promotes nuclear localization.

These findings suggest that specific nucleotide compositions and motif-associated physicochemical properties play a critical and class-dependent role in determining mRNA localization. Moreover, they validate the biological relevance of the proposed feature encoding strategy and highlight the interpretability of the model.

## Discussion

In this study, we proposed mRNALocator-imb, a heterogeneous ensemble learning framework that integrates a Random Forest (RF) model with a Transformer (TF) network for accurate prediction of mRNA subcellular localization. The framework leverages five complementary feature encoding strategies to capture both biologically interpretable and data-driven sequence characteristics. Specifically, four handcrafted feature representations (TPCP, Mismatch, RCKmer, and PseKNC) were fused to train the RF submodel, while k-mer–based representations were used to drive the TF submodel, enabling each component to exploit its respective strengths in modeling structured features and sequential dependencies.

To further enhance model robustness, we incorporated tailored imbalance-handling strategies for each submodel, including SMOTE for the RF branch and Random Oversampling combined with LDAM loss for the TF branch. Hyperparameters were systematically optimized using cross-validation–guided search, ensuring balanced and reliable performance across all classes.

Comprehensive experimental evaluations—including ten-fold cross-validation, systematic assessment of imbalance-handling strategies, and benchmarking against existing state-of-the-art methods on an independent test set—demonstrate that mRNALocator-imb consistently achieves superior performance across multiple evaluation metrics. These results confirm the effectiveness of integrating multi-scale feature representations with heterogeneous model architectures and imbalance-aware optimization.

Beyond predictive performance, we further enhanced the interpretability of the framework by applying SHapley Additive exPlanations (SHAP) to analyze feature contributions within the RF submodel. The results reveal that C/G-enriched sequence patterns and functional attributes of specific trinucleotide motifs (e.g., CCT) play a dominant and class-dependent role in determining mRNA localization. These findings not only validate the biological relevance of the proposed feature encoding strategies but also provide novel insights into potential sequence determinants underlying mRNA subcellular distribution.

Despite these advances, several limitations warrant further investigation. First, the current framework relies primarily on sequence-derived features and does not explicitly incorporate secondary or tertiary RNA structural information, which may contribute additional predictive power. Second, although the ensemble design improves performance, it increases model complexity and computational cost, which may limit scalability in large-scale applications. Third, while SHAP analysis provides valuable interpretability, further experimental validation is required to confirm the biological significance of the identified sequence motifs.

Future work will focus on integrating multi-modal data sources, such as RNA secondary structure, protein–RNA interaction information, and spatial transcriptomics data, to further enhance predictive accuracy and biological interpretability. In addition, more advanced representation learning strategies and lightweight model designs will be explored to improve scalability and deployment efficiency.

In summary, mRNALocator-imb provides a robust, interpretable, and imbalance-aware framework for mRNA subcellular localization prediction. The methodological design and biological insights presented in this study offer a generalizable paradigm for addressing imbalanced sequence classification problems in bioinformatics and may facilitate future advances in RNA biology and computational genomics.

## Data Availability

The original contributions presented in the study are included in the article/Supplementary Material, further inquiries can be directed to the corresponding authors.

## Footnotes

1 auto: The model automatically uses all input features.

2 sqrt: The square root of the total number of features is used.

3 log2: The $\log_{2}$ of the total number of features is used.
