# Supplementary material for: mRNALocator-imb: an imbalance-tolerant ensemble framework integrating random forest and transformer for mRNA subcellular localization prediction
Source: Front Genet. 2026 May 20;17:1861282. doi: 10.3389/fgene.2026.1861282 (PMC13229483; doi:10.3389/fgene.2026.1861282)
Supplement: Supplementary file 1 [file Supplementaryfile1.docx]

Supplementary Materials

## S1. Feature Encoding Strategies

To transform raw FASTA sequences into numerical representations suitable for computational modeling while preserving biologically relevant information, mRNALocator-imb employs five complementary feature encoding strategies. These approaches are categorized into two groups: manual (handcrafted) feature encoding and sequence-driven feature encoding. Together, they provide a multi-scale and biologically informed representation of mRNA sequences.

### Manual Feature Encoding

Manual feature encoding derives interpretable descriptors from nucleotide sequences based on established biological knowledge. In this study, four representative encoding schemes, including TPCP, Mismatch, RCKmer, and PseKNC, are integrated to capture physicochemical properties, compositional statistics, and sequence-order effects.

#### Trinucleotide Physicochemical Property (TPCP)

TPCP integrates intrinsic physicochemical properties of trinucleotide units with their occurrence frequencies, enabling joint characterization of sequence composition and functional biochemical attributes. Unlike conventional k-mer approaches that capture only frequency patterns, TPCP incorporates experimentally validated physicochemical properties (e.g., base stacking energy, melting temperature), thereby providing biologically interpretable features linked to molecular structure and function.

Formally, an RNA sequence length $L$ is defined as:

$RNA=\left( R_{1},R_{2},\ldots,R_{L} \right) , R_{i}\in\{A,T,C,G\}$ (1)

The sequence is decomposed into $L-2$ overlapping trinucleotides. The resulting feature vector is defined as:

$V_{TPCP}=[u_{1}f_{AAA}, u_{2}f_{AAC}, \ldots, u_{N}f_{TTT}]$ (2)

where the normalized frequency of each trinucleotide $f_{NNN}$ is calculated as:

$f_{NNN}=\frac{n_{NNN}}{L-2}$​​ (3)

where $u_{i}$represents the standardized value of the i-th physicochemical property. All physicochemical properties are normalized to zero mean and unit variance.

#### Mismatch k-mer (Mismatch)

The Mismatch method extends conventional k-mer encoding by allowing up to m mismatches in substring matching. This enables detection of conserved but variable sequence motifs, improving robustness to mutations.

For a given k-mer length $k$, let $\Omega_{k}$ denote the set of all possible k-mers. For each canonical k-mer $\alpha$, its $m$-neighborhood $N_{m}(\alpha)$ includes all k-mers differing by at most m mismatches.

The feature vector is defined as:

$V_{\mathrm{Mismatch}}=[f(m,k,\alpha_{1}), f(m,k,\alpha_{2}), \ldots, f(m,k,\alpha_{4^{k}})]$ (4)

where

$f(m,k,\alpha)=\frac{c\left( m,k,\alpha\right)}{L-k+1}$ (5)

and $c(m,k,\alpha)$​ denotes the number of substrings belonging to $N_{m}(\alpha)$.

In this study, the k-mer length was set to 5 (k = 5), and the maximum number of allowed mismatches was fixed at 1 (m=1), based on commonly adopted settings in bioinformatics sequence analysis and the results of preliminary parameter tuning.

#### Reverse Complement k-mer (RCKmer)

RCKmer accounts for reverse complementarity by merging counts of each k-mer and its reverse complement, reducing redundancy and capturing strand-invariant sequence features.

For a k-mer $\alpha$, its reverse complement $RC(\alpha)$ is defined based on Watson–Crick pairing rules. The feature vector is constructed over reverse complement clusters $\Theta_{k}$:

$V_{RCKmer}=[f(k,\theta_{1}), f(k,\theta_{2}), \ldots, f(k,\theta_{\left| \Theta_{k} \right|})]$ (6)

where

$f\left( k,\theta\right)=\frac{n_{\alpha}+n_{RC(\alpha)}}{L-k+1}$ ​​(7)

This representation improves generalization by encoding biologically equivalent sequence patterns.

#### Pseudo K-tuple Nucleotide Composition (PseKNC)

PseKNC extends k-mer composition by incorporating sequence-order information through physicochemical correlation factors. It captures both local composition and long-range dependencies.

The feature vector consists of two components:

k-tuple frequency vector:

$F_{KNC}=[f_{1}, f_{2}, \ldots, f_{4^{k}}]$ (8)

sequence-order correlation factors:

$\tau_{j}=\frac{1}{L-j}\sum_{i=1}^{L-j} J(R_{i},R_{i+j})$ (9)

where

$J(R_{i},R_{i+j})=\frac{1}{N}\sum_{\mu=1}^{N} [P_{\mu}(R_{i})-\overline{P_{\mu}}][P_{\mu}(R_{i+j})-\overline{P_{\mu}}]$ (10)

The final feature vector is:

$V_{\mathrm{PseKNC}}=[v_{1}, v_{2},\ldots, v_{4^{k}+\lambda}]$ (11)

where a weighting factor $\omega$ balances composition and correlation features.

The weighting factor was set to 0.1 (ω = 0.1), and the correlation length was fixed at 2 (λ = 2) as default values. These parameters were chosen based on commonly adopted settings in RNA sequence feature extraction and further validated through preliminary experiments to achieve a balance between local nucleotide composition and global sequence-order information.

### 1.2 Sequence Feature Encoding

Sequence-driven encoding captures intrinsic compositional patterns without relying on predefined biological knowledge.

1. k-mer Encoding

k-mer encoding represents RNA sequences as frequency distributions of substrings of length k, providing an alignment-free and scalable representation. It is based on the compositional similarity hypothesis, which assumes that sequences with similar k-mer profiles share functional or structural properties.

For a sequence of length $L$, the total number of k-mers is $L - k + 1$, and the normalized frequency of each k-mer $m$ is:

$f_{m}=\frac{C_{m}}{\sum_{i=1}^{4^{k}} C_{i}}$ ​(12)

where $C_{m}$​ denotes the occurrence count of k-mer $m$.

For the Transformer branch, only k-mers with k = 8 were used to generate frequency vectors as input features. In contrast, k values of 3, 4, and 5 were not employed in the Transformer branch, but were instead used for handcrafted feature encoding in the Random Forest branch: k = 5 for Mismatch, k = 4 for RCKmer, and k = 3 for PseKNC.

## S2. Evaluation Metrics

To comprehensively evaluate model performance under class imbalance, we employ Balanced Accuracy (BACC), Area Under the ROC Curve (AUC), and Macro F-measure.

### Balanced Accuracy (BACC)

BACC accounts for class imbalance by averaging recall across classes:

$BACC=\frac{1}{2}(\frac{TP}{TP+FN}+\frac{TN}{TN+FP})$ (13)

(2) Area Under the ROC Curve (AUC)

AUC evaluates the model’s discriminative ability across all classification thresholds: $AUC=\int_{0}^{1} TPR\left( FPR \right)d(FPR)$ (14)

(3) Macro F-measure

Macro F-measure evaluates performance equally across all classes:

$Precision(i)=\frac{TP\left( i \right)}{TP\left( i \right)+FP\left( i \right)}$ (15)

$Recall(i)=\frac{TP\left( i \right)}{TP\left( i \right)+FN(i)}$ ​(16)

$Macro F-measure=\frac{1}{m}\sum_{i=1}^{m} \frac{2\times Precision\left( i \right)\times Recall(i)}{Precision\left( i \right)+Recall\left( i \right)}$ ​(17)
